# Sociodemographic and health service organizational factors associated with the choice of the private versus public sector for specialty visits: Evidence from a national survey in Italy

**DOI:** 10.1371/journal.pone.0232827

**Published:** 2020-05-07

**Authors:** Davide Pianori, Elisa Maietti, Jacopo Lenzi, Mattia Quargnolo, Stefano Guicciardi, Kadjo Yves Cedric Adja, Maria Pia Fantini, Federico Toth

**Affiliations:** 1 Department of Biomedical and Neuromotor Sciences, University of Bologna, Bologna, Italy; 2 Department of Political and Social Sciences, University of Bologna, Bologna, Italy; Universita degli Studi di Ferrara, ITALY

## Abstract

**Introduction:**

Although Italy’s NHS is funded through general taxation, the private sector plays an important role in health service provision and financing. The aim of this paper was to identify the sociodemographic and health service organizational factors associated with the propensity to seek specialist care in the private sector.

**Materials and methods:**

Data were retrieved from the national Istat survey “Health conditions and use of health services” carried out in 2012–2013. We selected adults with a specialty visit in the previous 12 months in the four most frequent medical specialties: ophthalmology, cardiology, obstetrics/gynecology and orthopedics. The study outcome was the choice to use a private service. In order to investigate the determinants of private use, we adopted the socio-behavioral model by Andersen and Newman, making a distinction between sociodemographic and healthcare organizational factors. The associations with the outcome were analyzed using chi-squared test, t-test and multivariable logistic regression analysis.

**Results and discussion:**

Use of private care varied widely, from 26.3% for cardiology to 53.6% for obstetrics/gynecology. Females, patients with higher educational levels and patients with higher self-reported economic resources sought more frequently private healthcare for all specialties; younger patients and employed patients were more likely to seek private care for ophthalmic conditions. Exemption from copayment for public services reduced more than half the propensity to seek private care. Trust in this healthcare service was the main reason for private users (52.5%) followed by waiting time (26.7%) and physician choice (20.1%).

**Conclusion:**

The attitude of the population to use private services for specialist visits is linked both to sociodemographic and health services organizational factors: the former are unmodifiable while the latter are susceptible to managerial and health policy actions. In a public-financed, universal coverage system, policy makers may act upon the organizational factors that make private health facilities more attractive in order to reduce private care use.

## Introduction

The National Health Service (NHS) implemented in Italy is funded through general taxation. The NHS is committed to providing a basic package of “essential” healthcare services to the entire resident population. The so-called “essential levels of care” (Livelli Essenziali di Assistenza, LEAs) include most primary, outpatient specialist, hospital, emergency and preventive care services.

About two thirds of the healthcare services financed by the NHS are provided by public providers, while a third is provided by private providers under contract with the public service [1]. Patients can freely choose between NHS providers and contracted private suppliers (accreditated providers), without additional charges. For this reason, contracted private providers are assimilated to public suppliers.

Private healthcare spending accounts for 26% of the total health expenditure [2]. Private spending is 90% out-of-pocket, while the remaining 10% is used to buy private health insurance, which is either complementary or supplementary to the coverage provided by the NHS. Based on current estimates, approximately 22% of the Italian population has an additional private coverage [3].

Leaving aside the premiums paid for voluntary private insurance, private health expenditure in Italy essentially comprises three items.

The first consists of services excluded from the essential levels of care. Most dental procedures, outpatient physiotherapy and long-term care are not included in the LEAs. Individuals must pay out-of-pocket for the entire cost of services not envisaged by the LEAs.

The second includes the co-payments charged to patients. Primary care and hospitalizations are provided free of charge. Drugs, inappropriate attendance in emergency wards, specialist examinations and diagnostic procedures not consequent to hospitalization are provided upon co-payment by the patient. Co-payment varies depending on family income and region of residence. Individuals suffering from certain pathologies and low-income healthcare users are exempt from co-payment. On average, Italians spend about 26 euros per capita a year for co-payments to buy drugs, and about 22 euros for co-payments related to specialist outpatient services [4].

The third item consists of services included in the LEAs (and therefore formally guaranteed by the NHS) that Italians prefer to purchase from private providers.

In addition to their public practice, the medical staff of the Italian NHS are also allowed to work as private professionals. NHS physicians wishing to pursue their private practice can choose between two alternatives: the so-called *intra-moenia* and *extra-moenia*. *Intra-moenia* refers to private practice within the public facility where the physician is employed, whereas *extra-moenia* refers to an independent activity outside the NHS, performed in private facilities. Patients who decide to avail themselves of *intra-moenia* services are required to pay the full price of the consultation. This is the reason why intra-moenia practice may be considered private activity.

This paper is focused on the health services made available by the Italian NHS but that individuals prefer to seek in private facilities.

Previous research [1, 5–8] has highlighted that Italians choose the private sector for a number of reasons: 1) waiting times for private consultations are usually much shorter than those offered by public facilities; 2) patients may consider that certain private providers offer higher quality services than those guaranteed by the NHS; 3) patients may wish to choose a given health professional (this right is not envisaged in public facilities); 4) private facilities may be closer or more convenient to reach than public ones; 5) in some cases, the cost of a private consultation is comparable to the co-payment charged by the NHS.

If we set the Italian private health expenditure equal to 100, drugs account for about 41.8%, dental care for 20.2%, specialist examinations for 19%, diagnostic imaging procedures for 9.7%, lenses and glasses for 5.1%, and other expenses for 4.2% [3].

In this work, we will therefore investigate the propensity of Italians to seek private healthcare and pay for services they would be entitled to receive from the public sector. We will use the data collected by the Italian National Institute of Statistics, focusing particularly on specialist outpatient services. The purpose of this paper is to identify the sociodemographic and health service organizational factors associated with private specialist consultations.

## Materials and methods

Our data were retrieved from the national survey “Health conditions and use of health services” carried out in 2012–2013. This survey is part of the “Multipurpose Surveys on Households”, introduced in 1993 and conducted every five years by the Italian National Institute of Statistics (Istat) until 2013, when the questionnaire was reviewed to conform to the European standards. The survey consists of a self-administered paper questionnaire and a face-to-face interview with paper questionnaire. The collection methodology has been described in detail elsewhere [9–11].

A total of 49,811 households were sampled, and respondents to the questionnaire were 119,073. For this study, we selected adults (≥18 years of age) with a specialty visit over a lookback period of 12 months. The outcome of interest was the use of a private versus public facility for the last specialty visit. This information, together with medical specialty, was retrieved from two multiple-choice questions: (1) “Were you seen by a specialist for medical consultation in the last 12 months, and if so, what was the area of specialization of the doctor that visited you?” and (2) “Were you seen in a private facility or public/accreditated facility?”.

Considering the type of payment for the last specialty visit we excluded the intra-moenia activities from the analysis, in order to compare the private-private users (private facility and private funding) and the public-service users (NHS funding, including accreditated facilities). To investigate the determinants of private use we adopted the distinction made by Andersen and Newman [12] who proposed a socio-behavioral model that has been extensively used in studies investigating the use of health services [13]. We distinguished individual sociodemographic and illness level characteristics versus societal-organizational determinants of medical care utilization. We assumed that sociodemographic factors are hardly modifiable by decision makers, while organizational factors are directly affected by the managerial strategies and policies adopted.

We considered the following sociodemographic factors: age, sex, marital status (never married, married, separated/divorced, widowed), educational attainment (primary school or lower, middle school, high school, university degree or higher), occupation (job seeker, employed, student, housewife, retired from work, other condition) and self-rated economic resources (excellent, adequate, low, insufficient). Illness level was measured as the occurrence of chronic diseases in terms of multimorbidity (defined as the presence of ≥3 chronic conditions). To depict the characteristics of the health services delivery system and health policies we considered the answer to the following multiple-choice question “Why did you choose that type of facility?”. The respondent could pick one or more reasons (closeness, minor costs, trust in this healthcare service, physician choice possibility, shorter waiting time, and better conditions and time flexibility). Finally we explored, at individual level, the exemption from co-payment for public health services (not exempt, completely exempt) as a determinant highly influenced by health policies.

To control for regional differences that might impact the use of private or public facilities, the region of residence was also included in statistical analyses. Italy is subdivided into 20 regions: Piedmont, Aosta Valley, Lombardy, Liguria, Trentino-South Tyrol, Veneto, Friuli-Venezia Giulia, Emilia-Romagna, Tuscany, Umbria, Marche, Lazio, Abruzzo, Molise, Campania, Apulia, Basilicata, Calabria, Sicily, and Sardinia.

Since the early 90s, regional governments have been granted broad autonomy in planning and organizing healthcare services in their own territory. This means that the individual regional government is responsible for identifying hospitals to turn into hospital agencies and deciding how many local healthcare agencies to divide their territory into. The appointment of the general managers of the healthcare agencies is also the responsibility of the regional council and it is at regional level that the criteria for crediting and remunerating both public and private suppliers are established.

### Statistical analysis

Statistical analyses were restricted to the most frequent medical specialties observed in the study sample, namely ophthalmology, cardiology, obstetrics/gynecology and orthopedics. All analyses were stratified by these specialties.

Continuous variables were summarized as mean ± standard deviation; discrete and categorical variables were summarized as frequencies and percentages. Categories with low percentages (<5%) were collapsed to get more robust estimates. In particular, we merged students with housewives, and retired from work with the category “others”; we also merged excellent and adequate economic resources and, for obstetric/gynecological visits only, separated/divorced with widowed.

In order to identify the characteristics that distinguish private from public care users, first we compared the two groups using the chi-squared test or independent-sample *t*-test, as appropriate. Second, we carried out a multivariable logistic regression analysis including all the potential predictors described earlier. Categorical variables, including region of residence, were entered into the model as dummy variables. The goodness of fit of the model was evaluated with the Hosmer-Lemeshow test and the discriminative ability was assessed with the *C*-statistic.

Statistical significance level was set at 0.01. All analyses were performed using the Stata software package, version 15 (StataCorp. 2017. *Stata Statistical Software*: *Release 15*. College Station, TX: StataCorp LLC).

## Results

In the sample of 50,871 adults with a visit in the past 12 months, the most frequent specialties were ophthalmology (n = 8,619, 16.9%), cardiology (n = 7,932, 15.6%), obstetrics/gynecology (n = 7,858, 15.5%) and orthopedics (n = 7,290, 14.3%); other specialty visits were carried out by less than 5% of respondents. Globally the proportion of people who referred to a public facility in intra-moenia was 12.1% (ranging from 8.1% for dietetics to 15.5% for gastroenterology). The characteristics of the study patients with a visit in the four most frequent specialties, excluding intramoenia visits, are summarized in Table 1. About two thirds were female and mean age was 55.2±18.4 years. Multimorbidity was present in 1 out of 4 patients. Nearly 59% were married, 38% were employed and 62% declared adequate economic resources., Approximately 1 out of 5 reported to be totally exempt from co-payment for health services. Age and gender distribution widely differed across medical specialties: patients seen by a cardiologist were older (64.7±16.0) and more frequently male (56.5%), while obstetric/gynecological visits were more common among young women (42.7±13.1 years).

**Table 1:** Patients characteristics, overall and by medical specialty of the visit.

	Total (n = 27038)	Cardiology (n = 7049)	Orthopedics (n = 6212)	Ophthalmology (n = 7745)	Obstetrics-gynecology (n = 6932)
**Female, n (%)**	17631 (63.1)	3067 (43.5)	3491 (56.2)	4141 (53.5)	6932 (100)
**Age (years), mean ± SD**	55.2 ± 18.4	64.7 ± 16.0	57.1 ± 18.0	56.2 ± 18.7	42.7 ± 13.1
**Educational attainment, n (%)**					
Primary or lower	7610 (27.2)	2939 (41.7)	1987 (32.0)	2129 (27.5)	555 (8.0)
Middle school	7287 (26.1)	1784 (25.3)	1791 (28.8)	1898 (24.5)	1814 (26.2)
High school	9478 (33.9)	1752 (24.9)	1866 (30.0)	2760 (35.6)	3100 (44.7)
Degree or higher	3563 (12.8)	574 (8.1)	568 (9.1)	958 (12.4)	1463 (21.1)
**Marital status, n (%)**					
Never married	5632 (20.2)	814 (11.5)	1234 (19.9)	1778 (23.0)	1806 (26.0)
Married	16352 (58.5)	4276 (60.7)	3537 (56.9)	4358 (56.3)	4181 (60.3)
Separated/divorced	2167 (7.8)	443 (6.3)	484 (7.8)	542 (7.0)	698 (10.1)
Widow	3787 (13.6)	1516 (21.5)	957 (15.4)	1067 (13.8)	247 (3.6)
**Occupational status, n (%)**					
Employed	10586 (37.9)	1783 (25.3)	2284 (36.8)	2833 (36.6)	3686 (53.2)
Job seeker	2219 (7.9)	385 (5.5)	494 (7.9)	503 (6.5)	837 (12.1)
Housewife	5028 (18.0)	1276 (18.1)	1142 (18.4)	1178 (15.2)	1432 (20.7)
Student	1021 (3.7)	87 (1.2)	187 (3.0)	439 (5.7)	308 (4.4)
Retired from work	8193 (29.3)	3274 (46.4)	1896 (30.5)	2587 (33.4)	436 (6.3)
Other	891 (3.2)	244 (3.5)	209 (3.4)	205 (2.6)	233 (3.4)
**Self-rated income, n (%)**					
Excellent	514 (1.8)	120 (1.7)	115 (1.9)	151 (1.9)	128 (1.8)
Adequate	16870 (60.4)	4045 (57.4)	3597 (57.9)	4870 (62.9)	4358 (62.9)
Low	8996 (32.2)	2465 (35.0)	2127 (34.2)	2346 (30.3)	2058 (29.7)
Insufficient	1558 (5.6)	419 (5.9)	373 (6.0)	378 (4.9)	388 (5.6)
**Multimorbidity, n (%)**	7281 (26.1)	2991 (42.4)	1798 (28.9)	1771 (22.9)	721 (10.4)
**Exemption from copayment, n (%)**	6429 (23.0)	2538 (36.0)	1510 (24.3)	1878 (24.3)	503 (7.3)
**Area of residence, n (%)**					
North-West	6524 (23.4)	1402 (19.9)	1491 (24.0)	1863 (24.1)	1768 (25.5)
North-East	5742 (20.6)	1075 (15.2)	1304 (21.0)	1744 (22.5)	1619 (23.4)
Center	5376 (19.2)	1296 (18.4)	1193 (19.2)	1467 (18.9)	1420 (20.5)
South	7024 (25.1)	2247 (31.9)	1521 (24.5)	1805 (23.3)	1451 (20.9)
Islands	3272 (11.7)	1029 (14.6)	703 (11.3)	866 (11.2)	674 (9.7)

**Fig 1:**
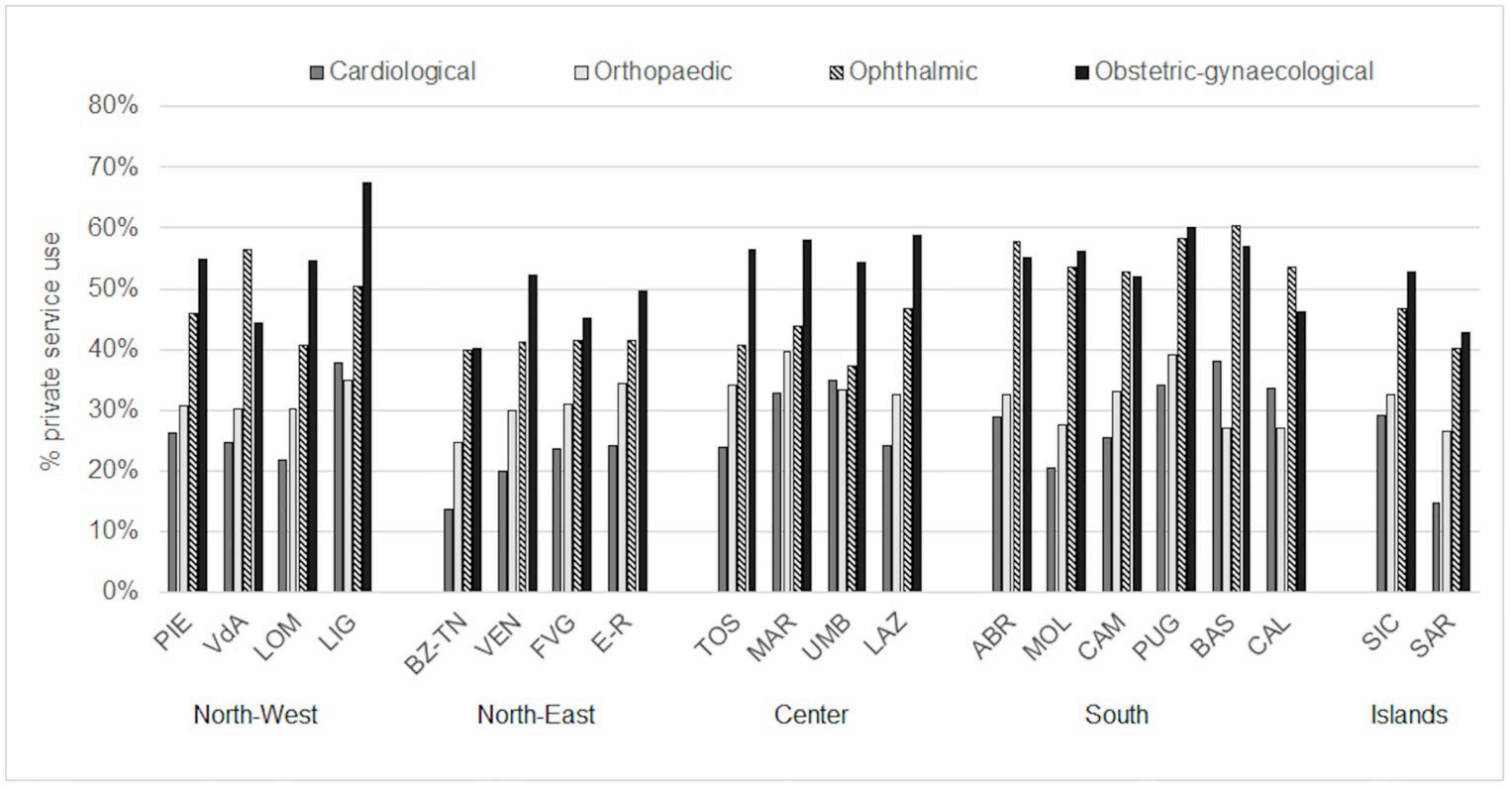
Private use rate for cardiologic, orthopedic, ophthalmic and obstetric-gynecological visits, across Italian regions (2013). PIE, Piedmont; VdA, Aosta Valley; LOM, Lombardy, LIG, Liguria; BZ-TN, Trentino-South Tyrol; VEN, Veneto, FVG, Friuli-Venezia Giulia; E-R, Emilia-Romagna; TOS, Tuscany, MAR, Marche, UMB, Umbria; LAZ, Lazio; ABR, Abruzzo, MOL, Molise, CAM, Campania, PUG, Apulia, BAS, Basilicata; CAL, Calabria; SIC, Sicily; SAR, Sardinia.

Use of private care exhibited a large variation across specialties, from 26.3% for cardiology and 32.0% for orthopedics to 46.1% for ophthalmology and 53.6% for obstetrics/gynecology. Differences across regions were also present (Fig 1): Trentino-South Tyrol and Sardinia had the lowest use of private service for all specialties, while Apulia, Basilicata and Liguria had the highest.

As shown in S1 Table, consistent among specialties, private service users were on average younger, had a higher education attainment, were more frequently employed, had more economic resources, had a lower number of concurrent chronic conditions and were less frequently exempt from co-payment.

Results of multivariable logistic regression analysis by medical specialty (ophthalmology, cardiology and orthopedics), are presented in Table 2: females were more likely to seek private care than males; younger patients and employed patients were more likely to seek private care for ophthalmic conditions; patients with higher educational levels resorted more frequently to private healthcare for all specialties. Multimorbidity and marital status were unrelated to private service use. Interestingly higher self-reported economic status showed a significant dose-response effect with the use of private care, while exemption from co-payment reduces by half the propensity to private use.

**Table 2:** Factors associated with use of private services for cardiologic, orthopedic and ophthalmic visits: Results of multivariable logistic regressions.

	Cardiologic (n = 7049)			Orthopedic (n = 6212)			Ophthalmic (n = 7745)		
	OR	99% IC	p-value	OR	99% IC	p-value	OR	99% IC	p-value
**Gender**			**0.008**			**<0.001**			**0.008**
Male	1.00	-		1.00	-		1.00	-	
Female	1.20	1.01–1.43		1.27	1.07–1.50		1.15	1.00–1.32	
**Age (years)**			0.131			0.265			**<0.001**
18–34 §	1.00	-		1.00	-		1.00	-	
35–49	0.94	0.63–1.40		1.14	0.85–1.54		0.67	0.52–0.85	
50–64	0.80	0.54–1.19		0.99	0.73–1.35		0.61	0.47–0.79	
65+	0.93	0.60–1.42		1.09	0.75–1.58		0.61	0.45–0.83	
**Educational attainment**			**<0.001**			**0.004**			**<0.001**
Primary or lower §	1.00	-		1.00	-		1.00	-	
Middle school	0.94	0.76–1.17		1.12	0.89–1.41		1.17	0.96–1.42	
High school	1.41	1.14–1.75		1.35	1.06–1.72		1.49	1.22–1.83	
Degree or higher	1.25	0.93–1.67		1.36	1.00–1.85		1.51	1.17–1.95	
**Marital status**			0.155			0.020			0.781
Never married §	1.00	-		1.00	-		1.00	-	
Married	1.22	0.93–1.60		1.17	0.93–1.48		1.01	0.83–1.23	
Separated/divorced	1.02	0.69–1.51		0.91	0.65–1.27		0.97	0.73–1.30	
Widow	1.21	0.88–1.67		0.98	0.71–1.35		1.09	0.83–1.44	
**Occupational status**			0.015			0.049			**<0.001**
Job seeker §	1.00	-		1.00	-		1.00	-	
Employed	1.54	1.06–2.25		1.23	0.91–1.67		1.67	1.26–2.20	
Housewife/student	1.27	0.83–1.92		0.98	0.70–1.38		1.28	0.95–1.72	
Retired from work or other	1.30	0.87–1.95		1.01	0.71–1.43		1.35	0.99–1.84	
**Self-rated income**			**<0.001**			**<0.001**			**<0.001**
Insufficient §	1.00	-		1.00	-		1.00	-	
Low	1.43	0.98–2.10		1.43	0.99–2.08		1.40	1.02–1.92	
Adequate or Excellent	2.13	1.47–3.10		2.01	1.40–2.90		1.82	1.33–2.48	
**Multimorbidity**			0.852			0.237			0.197
No §	1.00	-		1.00	-		1.00	-	
Yes	1.01	0.86–1.19		1.09	0.90–1.31		0.92	0.76–1.09	
**Exemption from copayment**			**<0.001**			**<0.001**			**<0.001**
Not exempt §	1.00	-		1.00	-		1.00	-	
Completely exempt	0.46	0.38–0.56		0.45	0.36–0.57		0.49	0.40–0.59	

^§^ Reference category.

OR and 99% CI estimates are adjusted for region of residence; p-value refer to likelihood ratio test between multivariable nested models (with and without the variable).

As for obstetric/gynecological visits (Table 3), we found that higher educational attainment, higher income and being employed were significantly associated with a higher use of private care, while being exempt from copayment and being separated/divorced or widowed was associated with a lower use. The odds ratios associated with the region of residence are reported in the S2 Table. All the models had an acceptable goodness of fit (Hosmer-Lemeshow test p-value>0.1) but a low discriminative ability (*C*-statistic<0.7).

**Table 3:** Factors predisposing to the use of private services for obstetric-gynecological visits: Results of multivariable logistic regression model.

	Obstetric-gynecological (n = 6392)		
	OR	99% IC	p-value
**Age (years)**			0.204
18–30 §	1.00	-	
31–45	0.90	0.74–1.11	
46–60	0.83	0.67–1.04	
>60	0.83	0.59–1.18	
**Educational attainment**			**<0.001**
Primary or lower §	1.00	-	
Middle school	1.51	1.12–2.03	
High school	1.87	1.38–2.52	
Degree or higher	2.05	1.48–2.83	
**Marital status**			**0.004**
Never married §	1.00	-	
Married	0.97	0.82–1.15	
Separated/divorced or widow	0.76	0.60–0.96	
**Occupational status**			**<0.001**
Job seeker §	1.00	-	
Employed	1.35	1.08–1.68	
Housewife/student	1.04	0.82–1.32	
Retired from work or other	0.95	0.70–1.32	
**Self-rated income**			**<0.001**
Insufficient §	1.00	-	
Low	1.61	1.18–2.21	
Adequate or Excellent	2.44	1.78–3.33	
**Multimorbidity**			0.779
No §	1.00	-	
Yes	1.03	0.82–1.29	
**Exemption from copayment**			**<0.001**
Not exempt §	1.00	-	
Completely exempt	0.41	0.31–0.56	

^§^Reference category

OR and 99% CI estimates are adjusted for the region of residence; p-value refer to the likelihood ratio test between multivariable nested models (with and without the variable).

**Fig 2:**
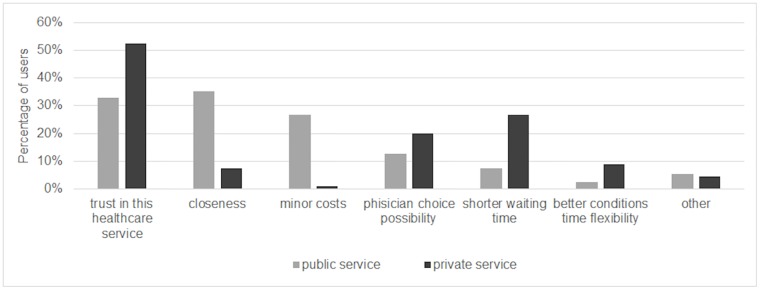
Reasons endorsed for the choice of public or private sector.

Fig 2 shows the proportions of the reasons given for the choice of the private or the public sector. Trust in the private healthcare service was the main reason for private users (52.5%) followed by waiting time (26.7%) and physician choice (20.1%). Public users reported trust in the healthcare service as well as closeness in 1 out of 3 cases, while minor costs were reported in 26.9% of cases. As to the reasons for the private service choice, the proportions varied significantly across specialties (S3 Table). Trust in the healthcare services was the main reason for all specialties, although it was more common among obstetrics-gynecology private users (57.6%) and less common among cardiology private users (46.8%); waiting time was the second reason, except for obstetrics-gynecology, where the second reason was the possibility of choosing the physician.

## Discussion

Italy is a country with a high propensity to seek specialist care resort in the private sector. Our results indicate that more than 40% of specialist examinations are paid out-of-pocket, most of which performed by private providers (30%). We should point out that these levels of recourse to private services are consistent with two previous studies based on the same data [14, 15], but are markedly lower than those of other research studies carried out in Italy. A recent survey [3] estimated that over 50% of specialist examinations in Italy are provided by the private sector. In a previous survey by the National Institute of Statistics 48% of specialist examinations were performed privately and fully paid by patients [5, 8].

In our study, the use of the private sector varies considerably depending on the medical specialty: 26.3% of cardiac examinations and 32.0% of orthopedic examinations are performed privately; much higher percentages are reported for ophthalmological (46.1%) and obstetric-gynecological examinations (53.6%).

An explanation of this finding is the patients’ desire to be followed over time by the same trusted specialist. Ophthalmological, obstetric and gynecological examinations are frequently routine check-ups, which patients undergo periodically—more so than orthopedic and cardiologic consultations. For such examinations, it is hence understandable that patients prefer to be followed over time by the same physician, with whom they establish a relationship of confidence and trust. As mentioned above, patients can choose the facility within the NHS where they want to be examined (or hospitalized), but they are not free to choose the physician. Conversely, the choice of a given physician is guaranteed in the private sector.

Orthopedic and cardiologic examinations are instead more sporadic, do not necessarily require the establishment of a stable relationship with the specialist and are linked to individual health problems. Indeed, as reported in our results, cardiology and orthopedics patients choose private service to receive the consultation in a trusted facility but also to avoid long waiting times.

Concerning the sociodemographic factors that may influence the extent to which people use the private healthcare sector, we identified four factors: family income, educational level, being employed, female gender.

Results concerning family income and educational level are consistent with previous research, involving both Italy [7, 16–18] and other countries where a NHS is implemented, showing that the probability of using private care [19–22] and taking out a private health insurance [23–27] increases with income and education.

The other two factors (i.e., being employed and female gender) that also emerged as being relevant in a previous research [14, 21], are perhaps less easy to interpret.

As to the employment factor: other things being equal (e.g., income or education level), the most plausible explanation for the higher use of private facilities among employed people is that services booked through the NHS cause some inconvenience for the user, some “transaction costs”, that are not envisaged by private services, regardless of whether the individual is subject to co-payment or not. First, a formal referral by the family doctor is required to book a specialist examination or diagnostic procedure within the NHS. In the private sector, the family doctor’s referral is not necessary. To schedule an appointment within the NHS, the patient has to contact the CUP (*Centro Unico di Prenotazione*, or single booking center) of the public service: in many cases, this involves going in person to a local health office, at given opening hours, and queuing up at the counter. A phone call is usually enough to book a private appointment. Also, private appointments usually allow for greater flexibility in terms of hours: it is possible to book an appointment in the afternoon or evening, outside the normal working hours. This flexibility is not envisaged in most public facilities. All these factors make it more convenient for a worker to set an appointment privately.

As to the gender, one possible reason of our finding that women are more likely to turn to the private sector is that they may be more inclined to disclose their symptoms and establish an ongoing relationship with the healthcare professionals. Therefore, the possibility offered by the private sector to be followed by the same professional over time may play an important role.

Considering the exemption from co-payment the results does not require special explanations. In fact we saw that individuals who are exempt from co-payment—and therefore can undergo examinations and procedures provided by the NHS at no charge—are more likely to use the public service. This result is supported by a previous Italian study [14].

### Strengths and limitations

The main strength of our study is that it is based on a large and representative sample of the Italian population, based on a national survey conducted after the economic crisis. We acknowledge that these data could appear outdated, however this survey investigates much more domains as compared to more recent European surveys (e.g., EHIS 2015). Some limitations deserve to be commented: (1) we investigated only the last visit of the year, so the share of utilization of private services may be underestimated; (2) multivariable regression analysis, which included only sociodemographic characteristics and the exemption from copayment as explanatory variables, showed low discriminatory ability, suggesting that health service organizational factors might be the main drivers for the choice to seek specialist care in the private sector; (3) this is not the first Italian study to analyze the determinants of private care seeking for specialty visits [14–15], however is the first to investigate the determinants for each specialty.

## Conclusions

The attitude of the population to use private services for specialist visits is linked both to sociodemographic factors (age, gender, income, occupation and educational attainment) and to health services organizational factors (copayment, waiting time, freedom to choose the physician). What policy implications can thus be drawn from these results? In the short run, policy makers can only intervene on modifiable, organizational factors and italian regional governments may act upon the factors that make private health facilities more attractive in order to reduce private care use in a public-financed, universal coverage system. To this end, they could make booking procedures more user-friendly, allow patients to select the physician of their choice, and reform the co-payment system.

## Supporting information

S1 Table Comparison between public and private health services users for cardiologic, orthopedic, ophthalmic and obstetric-gynecological visits. (DOCX)

S2 Table Logistic regression model estimates for Italian regions (cardiologic, orthopedic, ophthalmic and obstetric-gynecological visits). (DOCX)

S3 Table Reasons endorsed for the choice of private sector for cardiologic, orthopedic, ophthalmic and obstetric-gynecological visits. (DOCX)
