# Supplementary material for: Sociodemographic and health service organizational factors associated with the choice of the private versus public sector for specialty visits: Evidence from a national survey in Italy
Source: PLoS One. 2020 May 7;15(5):e0232827. doi: 10.1371/journal.pone.0232827 (PMC7205245; doi:10.1371/journal.pone.0232827)
Supplement: S1 Table — (DOCX) [file pone.0232827.s001.docx]

**Table S1. Comparison between public and private health services users for cardiologic, orthopedic, ophthalmic and obstetric-gynecological visits**

|  | **Cardiologic** | | | **Orthopedic** | | | **Ophthalmic** | | | **Obstetric-gynecological** | | |
| --- | --- | --- | --- | --- | --- | --- | --- | --- | --- | --- | --- | --- |
|  | **Public** | **Private** | **p-value** | **Public** | **Private** | **p-value** | **Public** | **Private** | **p-value** | **Public** | **Private** | **p-value** |
|  | **(n= 5192)** | **(n= 1857)** |  | **(n= 4227)** | **(n= 1985)** |  | **(n= 4172)** | **(n= 3573)** |  | **(n=3218)** | **(n= 3714)** |  |
| **Female, n (%)** | 2250 (43.3) | 817 (44.0) | 0.623 | 2372 (56.1) | 1119 (56.4) | 0.849 | 2248 (53.9) | 1893 (53.0) | 0.427 | 3218 (100) | 3714 (100) | - |
| **Age (years), mean ± SD** | 65.2 ± 15.8 | 63.0 ± 16.4 | **<0.001** | 58.2 ± 18.3 | 54.8 ± 17.1 | **<0.001** | 59.5 ± 18.1 | 52.3 ± 18.7 | **<0.001** | 43.9 ± 14.0 | 41.6 ± 12.2 | **<0.001** |
| **Educational attainment, n (%)** |  |  | **<0.001** |  |  | **<0.001** |  |  | **<0.001** |  |  | **<0.001** |
| Primary or lower | 2291 (44.1) | 648 (34.9) |  | 1498 (35.4) | 489 (24.6) |  | 1441 (34.5) | 688 (19.3) |  | 384 (11.9) | 171 (4.6) |  |
| Middle school | 1374 (26.5) | 410 (22.1) |  | 1228 (29.1) | 563 (28.4) |  | 1082 (25.9) | 816 (22.8) |  | 954 (29.7) | 860 (23.2) |  |
| High school | 1149 (22.1) | 603 (32.5) |  | 1165 (27.6) | 701 (35.3) |  | 1241 (29.7) | 1519 (42.5) |  | 1320 (41.0) | 1780 (47.9) |  |
| Degree or higher | 378 (7.3) | 196 (10.5) |  | 336 (7.9) | 232 (11.7) |  | 408 (9.8) | 550 (15.4) |  | 560 (17.4) | 903 (24.3) |  |
| **Marital status, n (%)** |  |  | 0.043 |  |  | **<0.001** |  |  | **<0.001** |  |  | **<0.001** |
| Never married | 605 (11.7) | 209 (11.2) |  | 844 (20.0) | 390 (19.7) |  | 807 (19.3) | 971 (27.2) |  | 783 (24.3) | 1023 (27.6) |  |
| Married | 3101 (59.7) | 1175 (63.3) |  | 2320 (54.9) | 1217 (61.3) |  | 2393 (57.4) | 1965 (55.0) |  | 1918 (59.6) | 2263 (60.9) |  |
| Separated/divorced | 332 (6.4) | 111 (6.0) |  | 339 (8.0) | 145 (7.3) |  | 286 (6.9) | 256 (7.2) |  | 374 (11.6) | 324 (8.7) |  |
| Widow | 1154 (22.2) | 362 (19.5) |  | 724 (17.1) | 233 (11.7) |  | 686 (16.4) | 381 (10.6) |  | 143 (4.4) | 104 (2.8) |  |
| **Occupational status, n (%)** |  |  | **<0.001** |  |  | **<0.001** |  |  | **<0.001** |  |  | **<0.001** |
| Job seeker | 308 (5.9) | 77 (4.1) |  | 356 (8.4) | 138 (6.9) |  | 286 (6.9) | 217 (6.1) |  | 445 (13.8) | 392 (10.6) |  |
| Employed | 1185 (22.8) | 598 (32.2) |  | 1401 (33.1) | 883 (44.5) |  | 1236 (29.6) | 1597 (44.7) |  | 1490 (46.3) | 2196 (59.1) |  |
| Housewife/student | 1026 (19.8) | 337 (18.2) |  | 946 (22.4) | 383 (19.3) |  | 893 (21.4) | 724 (20.3) |  | 892 (27.7) | 848 (22.8) |  |
| Retired from work or other | 2673 (51.5) | 845 (45.5) |  | 1524 (36.1) | 581 (29.3) |  | 1757 (42.1) | 1035 (28.9) |  | 391 (12.2) | 278 (7.5) |  |
| **Self-rated income, n (%)** |  |  | **<0.001** |  |  | **<0.001** |  |  | **<0.001** |  |  | **<0.001** |
| Adequate or Excellent | 2897 (55.8) | 1268 (68.3) |  | 2376 (56.2) | 1336 (67.3) |  | 2525 (60.5) | 2496 (69.9) |  | 1852 (57.6) | 2634 (70.9) |  |
| Low | 1942 (37.4) | 523 (28.2) |  | 1551 (36.7) | 576 (29.0) |  | 1396 (33.5) | 950 (26.6) |  | 1103 (34.3) | 955 (25.7) |  |
| Insufficient | 353 (6.8) | 66 (3.5) |  | 300 (7.1) | 73 (3.7) |  | 251 (6.0) | 127 (3.5) |  | 263 (8.2) | 125 (3.4) |  |
| **Multimorbidity, n (%)** | 2278 (43.9) | 713 (38.4) | **<0.001** | 1277 (30.2) | 521 (26.2) | **0.001** | 1135 (27.2) | 636 (17.8) | **<0.001** | 398 (12.4) | 323 (8.7) | **<0.001** |
| **Exemption from copayment, n (%)** | 2116 (40.8) | 422 (22.7) | **<0.001** | 1237 (29.3) | 273 (13.8) | **<0.001** | 1377 (33.0) | 501 (14.0) | **<0.001** | 381 (11.8) | 122 (3.3) | **<0.001** |
