# Supplementary material for: Sociodemographic and health service organizational factors associated with the choice of the private versus public sector for specialty visits: Evidence from a national survey in Italy
Source: PLoS One. 2020 May 7;15(5):e0232827. doi: 10.1371/journal.pone.0232827 (PMC7205245; doi:10.1371/journal.pone.0232827)
Supplement: S2 Table — (DOCX) [file pone.0232827.s002.docx]

**Table S2. Logistic regression model estimates for Italian regions (cardiologic, orthopedic, ophthalmic and obstetric-gynecological visits)**

|  | **Cardiologic (n= 7049)** | | **Orthopedic (n= 6212)** | | **Ophthalmic (n= 7745)** | | **Obstetric-gynecological (n= 6932)** | |
| --- | --- | --- | --- | --- | --- | --- | --- | --- |
|  | **OR** | **99% IC** | **OR** | **99% IC** | **OR** | **99% IC** | **OR** | **99% IC** |
| Trentino-South Tyrol | 1.00 | - | 1.00 | - | 1.00 | - | 1.00 | - |
| Piedmont | **2.49** | **1.13 - 5.47** | 1.39 | 0.84 - 2.29 | 1.38 | 0.92 - 2.08 | **2.12** | **1.44 - 3.12** |
| Aosta Valley | 2.17 | 0.79 - 5.96 | 1.30 | 0.65 - 2.62 | **2.05** | **1.05 - 3.98** | 1.16 | 0.64 - 2.10 |
| Lombardy | 1.71 | 0.79 - 3.68 | 1.29 | 0.81 - 2.06 | 1.08 | 0.73 - 1.60 | **1.88** | **1.32 - 2.67** |
| Liguria | **4.17** | **1.84 - 9.42** | **1.80** | **1.06 - 3.05** | **1.73** | **1.08 - 2.78** | **3.94** | **2.54 - 6.12** |
| Veneto | 1.57 | 0.70 - 3.49 | 1.37 | 0.83 - 2.25 | 1.14 | 0.76 - 1.74 | **1.84** | **1.26 - 2.68** |
| Friuli-Venezia Giulia | 2.11 | 0.89 - 5.00 | 1.44 | 0.83 - 2.49 | 1.16 | 0.74 - 1.81 | 1.34 | 0.86 - 2.09 |
| Emilia-Romagna | 2.09 | 0.94 - 4.62 | **1.72** | **1.04 - 2.83** | 1.18 | 0.77 - 1.80 | **1.57** | **1.07 - 2.30** |
| Toscana | 2.07 | 0.92 - 4.64 | 1.65 | 0.99 - 2.75 | 1.25 | 0.81 - 1.93 | **2.17** | **1.45 - 3.26** |
| Umbria | **3.46** | **1.49 - 8.07** | 1.71 | 0.93 - 3.12 | 1.04 | 0.62 - 1.76 | **2.00** | **1.21 - 3.31** |
| Marche | **3.33** | **1.47 - 7.55** | **2.35** | **1.36 - 4.07** | 1.41 | 0.88 - 2.26 | **2.35** | **1.50 - 3.68** |
| Lazio | 2.15 | 0.99 - 4.66 | 1.53 | 0.94 - 2.50 | 1.41 | 0.92 - 2.15 | **2.42** | **1.66 - 3.52** |
| Abruzzo | **2.86** | **1.25 - 6.53** | 1.54 | 0.87 - 2.72 | **2.33** | **1.42 - 3.81** | **2.13** | **1.32 - 3.44** |
| Molise | 1.75 | 0.71 - 4.29 | 1.42 | 0.70 - 2.91 | **2.13** | **1.17 - 3.86** | **2.40** | **1.34 - 4.29** |
| Campania | **2.21** | **1.03 - 4.74** | 1.64 | 1.00 - 2.68 | **1.79** | **1.17 - 2.73** | **2.15** | **1.44 - 3.20** |
| Puglia | **3.86** | **1.79 - 8.31** | **2.44** | **1.47 - 4.04** | **2.69** | **1.74 - 4.16** | **2.97** | **1.95 - 4.52** |
| Basilicata | **4.24** | **1.84 - 9.77** | 1.39 | 0.71 - 2.71 | **2.64** | **1.54 - 4.52** | **2.67** | **1.48 - 4.82** |
| Calabria | **3.84** | **1.74 - 8.49** | 1.38 | 0.77 - 2.45 | **2.13** | **1.33 - 3.42** | **1.92** | **1.17 - 3.14** |
| Sicilia | **2.97** | **1.38 - 6.40** | **1.74** | **1.05 - 2.88** | **1.57** | **1.02 - 2.40** | **2.33** | **1.57 - 3.46** |
| Sardegna | 1.25 | 0.55 - 2.83 | 1.28 | 0.74 - 2.20 | 1.17 | 0.74 - 1.83 | 1.33 | 0.82 - 2.18 |

Note: Trentino-South Tyrol was chosen as reference class; OR and 99% CI estimates were adjusted for age, sex, educational attainment, marital status, occupation, self-rated economic resources, multimorbidity and exemption from co-payment for public health services.
