# Supplementary material for: Sociodemographic and health service organizational factors associated with the choice of the private versus public sector for specialty visits: Evidence from a national survey in Italy
Source: PLoS One. 2020 May 7;15(5):e0232827. doi: 10.1371/journal.pone.0232827 (PMC7205245; doi:10.1371/journal.pone.0232827)
Supplement: S3 Table — (DOCX) [file pone.0232827.s003.docx]

**S3 Table. Reasons endorsed for the choice of private sector for cardiologic, orthopedic, ophthalmic and obstetric-gynecological visits**

|  | **Total  private users (N=11129)** | **Obstetric-gynecological private users (N=3714)** | **Ophthalmic private users (N=3573)** | **Orthopedic private users (N=1985)** | **Cardiologic private users (N=1857)** |  |
| --- | --- | --- | --- | --- | --- | --- |
|  | **n ( %)** | **n ( %)** | **n ( %)** | **n ( %)** | **n ( %)** | **p-value** |
| Trust in this health service | 5839 (52.5) | 2140 (57.6) | 1869 (52.3) | 961 (48.4) | 869 (46.8) | <0.001 |
| Shorter waiting time | 2967 (26.7) | 671 (18.1) | 1002 (28.0) | 674 (34.0) | 620 (33.4) | <0.001 |
| Physician choice possibility | 2240 (20.1) | 885 (23.8) | 609 (17.0) | 356 (17.9) | 390 (21.0) | <0.001 |
| Closeness | 812 (7.3) | 187 (5.0) | 295 (8.3) | 167 (8.4) | 163 (8.8) | <0.001 |
| Better conditions and time flexibility | 974 (8.8) | 373 (10.0) | 317 (8.9) | 156 (7.9) | 128 (6.9) | <0.001 |
| Other motivations | 492 (4.4) | 181 (4.9) | 142 (4.0) | 84 (4.2) | 85 (4.6) | 0.288 |
